# *Latilactobacillus curvatus* DCF0620 and postbiotics derived from soybean germ reduce colitis severity by modulating fibrosis and gut dysbiosis

**DOI:** 10.3389/fimmu.2025.1726298

**Published:** 2026-01-06

**Authors:** Jin-Sil Park, Hye Yeon Kang, JeongWon Choi, Wonjong Lee, Hyunbin Seong, Sang Hee Cho, Su Beom Lee, Nam Soo Han, Jaekwon Choi, Bo-In Lee, Mi-La Cho

**Affiliations:** 1 Rheumatism Research Center, Catholic Research Institute of Medical Science, College of Medicine, The Catholic University of Korea, Seoul, Republic of Korea; 2 Lab of Translational ImmunoMedicine, Catholic Research Institute of Medical Science, College of Medicine, The Catholic University of Korea, Seoul, Republic of Korea; 3 Department of Pathology, College of Medicine, The Catholic University of Korea, Seoul, Republic of Korea; 4 Department of Medical Sciences, Graduate School of The Catholic University of Korea, Seoul, Republic of Korea; 5 Central Research Institute, Dr. Chung’s Food Co. Ltd., Cheongju, Republic of Korea; 6 Brain Korea 21 Center for Smart GreenBio Convergence and Sustainable Regional Development, Division of Animal, Horticultural, Food Sciences, Chungbuk National University, Cheongju, Republic of Korea; 7 Divisions of Gastroenterology and Department of Internal Medicine, Seoul St. Mary’s Hospital, College of Medicine, The Catholic University of Korea, Seoul, Republic of Korea

**Keywords:** fibrosis, gut microbiota, inflammatory bowel disease, inflammation, *Latilactobacillus curvatus*, postbiotics

## Abstract

**Background:**

Given that microbiota dysbiosis is closely linked to the initiation and progression of inflammatory bowel disease (IBD), extensive research is underway to utilize beneficial probiotics as a therapeutic strategy for IBD. In this study, we compared the therapeutic efficacy of *Lacticaseibacillus paracasei* (*L. paracasei*) DCF0420, *Lactiplantibacillus plantarum* (*L. plantarum*) DCF0514, and *Latilactobacillus curvatus* (*L. curvatus*) DCF0620 and also investigated the potential of soybean germ-based postbiotics from DCF0620 as a treatment for IBD.

**Methods:**

Live *L. paracasei* DCF0420, *L. plantarum* DCF0514, *L. curvatus* DCF0620, or postbiotics were orally administered to DSS-induced colitis mice starting 7 days before DSS injection until the end of the experiment. Weight changes and the disease activity index were evaluated to assess disease severity. Inflammation and fibrosis were analyzed pathologically in intestinal tissue. Fecal samples from mice injected with postbiotics were subjected to 16S rRNA gene sequencing. Cytokine levels in spleen cell culture supernatants were measured by ELISA, and fibronectin levels in CCD-18Co cells were analyzed by Western blot.

**Results:**

Treatment with *L. paracasei* DCF0420, *L. plantarum* DCF0514, or *L. curvatus* DCF0620 increased the production of IL-10 in murine splenocytes in a dose-dependent manner under stimulation with anti-CD3 antibody or LPS. Furthermore, administration of each probiotic strain reduced the levels of fibronectin induced by TGF-β in CCD-18Co cells. In the DSS-induced colitis murine model, administration of *L. curvatus* DCF0620 effectively attenuated disease severity. Mechanistically, *L. curvatus* DCF0620 treatment controlled the infiltration of cells that express pro-inflammatory cytokines (TNF-α, IL-1β, IL-6, and IL-17) and fibrotic markers (TGF-β, Col1, and α-SMA) into the intestinal tissue of DSS-induced colitis mice. Notably, administration of soybean germ-based postbiotics derived from *L. curvatus* DCF0620 also demonstrated therapeutic efficacy in the DSS-induced colitis mice, specifically by improving the dysbiosis observed in the colitis mice.

**Conclusion:**

Our findings suggest that *L. curvatus* DCF0620 and its soybean germ-based postbiotics represent promising therapeutic agents for IBD by demonstrating potent anti-inflammatory, anti-fibrotic, and gut microbiota-modulating effects.

## Introduction

Inflammatory bowel disease (IBD) is a chronic and progressive disorder of the gastrointestinal tract characterized by relapsing intestinal inflammation that involves a complex interplay of genetic, environmental, microbial, and immune factors (1–3). It includes two major clinical conditions: Ulcerative Colitis, which affects the mucosal layer of the large intestine in a continuous and diffuse pattern, and Crohn’s Disease, which causes transmural chronic inflammation across various regions of the gastrointestinal tract (4, 5). Its pathogenesis remains incompletely understood but has been linked to impaired intestinal mucosal barrier function, abnormalities in intestinal epithelial and immune cells, and gut microbiota dysbiosis (4, 5). Currently, the main treatment interventions include immunosuppressants, glucocorticoids, 5-aminosalicylic acid, and immunomodulators (6). However, these drugs do not fundamentally repair mucosal damage, reduce gut inflammation, or restore barrier function, and they often cause side effects such as fatigue, nausea, headaches, and diarrhea (7). Moreover, long-term use of these drugs may lead to adverse outcomes such as drug resistance, potential toxicity, and reduced immune resilience in patients (8). Therefore, there is a need to develop new therapeutic strategies that minimize side effects while restoring gut barrier integrity and controlling inflammation.

The host’s gastrointestinal tract harbors a vast and diverse community of microbes, and growing evidence suggests that gut microbiota dysbiosis is closely associated with the onset of various diseases, particularly gastrointestinal disorders (9–11). Studies have shown a marked reduction in microbial diversity and significant shifts in microbial composition in patients with IBD compared to healthy controls (12). Therapeutic interventions such as the oral administration of probiotics or fecal microbiota transplantation have been shown to restore microbial balance and support gut homeostasis (13, 14).

Given that the loss of beneficial bacteria contributes to persistent intestinal inflammation in IBD, probiotics are gaining attention as a promising therapeutic strategy for a range of diseases, including IBD. Probiotics can mitigate chronic inflammation by scavenging reactive oxygen species, enhancing the host’s antioxidant defenses, and regulating the microbial composition of the gut (15–17). Among the most extensively studied probiotic genera are lactic acid bacteria (LAB), including *Lacticaseibacillus*, *Lactiplantibacillus*, and *Bifidobacterium* species (18). In the context of IBD, LAB inhibit pathogenic growth in the gut epithelium through competitive exclusion and enhance epithelial barrier function by promoting the production of tight junction proteins and antimicrobial peptides (19, 20). Additionally, they contribute to the attenuation of inflammatory responses by producing short-chain fatty acids (SCFAs) such as acetate and butyrate (18). While the anti-colitic effects of common LAB are well-established, the therapeutic potential of specific strains remains largely uncharacterized. For instance, *Latilactobacillus curvatus* (*L. curvatus*), a strain isolated from fermented foods, has only been investigated in a very limited number of studies regarding its role in IBD models (21, 22). Furthermore, the reported mechanism of action for *L. curvatus* has primarily focused on basic inflammation control, such as modulating the production of proinflammatory cytokines (e.g., interleukin (IL)-6 and tumor necrosis factor (TNF)-α. Crucially, the ability of *L. curvatus* to address critical long-term complications, such as intestinal fibrosis, remains virtually unexplored. This scarcity of research underscores the urgent need to thoroughly investigate the comprehensive therapeutic mechanisms of promising, specific strains like *L. curvatus*.

Postbiotics, defined as preparations of inanimate microorganisms and/or their components that confer health benefits to the host, have emerged as a complementary or alternative approach to probiotics (23). These bioactive compounds, including SCFAs, organic acids, bacteriocins, and microbial metabolites, have functional effects on the gut environment (24). Recent studies suggest that postbiotics may offer a safer and potentially more effective therapeutic option than probiotics due to their ability to modulate both the immune system and the gut microbiome without the risks associated with live microbial administration (25, 26).

In this study, we compared the therapeutic efficacy of *Lacticaseibacillus paracasei* (*L. paracasei*) DCF0420, *Lactiplantibacillus plantarum* (*L. plantarum*) DCF0514, and *L. curvatus* DCF0620, isolated from traditional Korean fermented foods, in mice with dextran sodium sulfate (DSS)-induced colitis. Furthermore, this study highlights a unique approach utilizing the heat-inactivated whole culture (including microbial cells and fermented soybean germ milk supernatant) of *L. curvatus* DCF0620 as a soybean germ-based postbiotic. Notably, the therapeutic efficacy of this postbiotic was associated with a significant improvement in gut microbiota dysbiosis observed in the DSS-colitis model, suggesting its potential as a novel therapeutic strategy for colitis.

## Materials and methods

### Isolation of microorganisms

Five samples of traditional Korean fermented foods were collected from Cheongju, Korea. For the isolation of LABs, 25 g of each spontaneously fermented sample was aseptically transferred to sterile plastic pouches and homogenized with a ten-fold volume of sterile 0.85% NaCl solution for 90 s. Following a tenfold serial dilution, the samples were inoculated onto De Man, Rogosa and Sharpe (MRS) agar plates. LAB strains were incubated anaerobically at 37°C. After 48 h of incubation, colonies with distinct morphologies were randomly selected and streaked onto fresh MRS agar plates. To identify the isolates, polymerase chain reaction amplification of the 16S ribosomal ribonucleic acid (rRNA) gene was performed using universal primers 27F (5′-AGAGTTTGATCMTGGCTCAG-3′) and 1492R (5′-GGTTACCTTGTTACGACTT-3′).

### Production of postbiotics via fermentation of soy germ

Soybean germs were obtained from Dr. Chung’s Food Co. Ltd. (Cheongju, Korea). Soy germ milk was prepared by wet grinding, followed by fiber separation. Glucose (3%, w/v) was added to the soy germ milk prior to fermentation. The initial pH of the broth was adjusted to 7.0 and sterilized at 121 °C for 15 min in an autoclave. The sterilized broth was inoculated with the tested strain culture (1%, v/v) and incubated at 37 °C for 24 h. After fermentation, the broth was heat-inactivated at 121 °C for 15 min. Sterility was confirmed by quantitatively testing the heat-inactivated broth for total aerobic bacteria, *E. coli*, coliforms, and lactic acid bacteria using plate count methods; all tested groups were confirmed to be sterile. Chemical characterization was performed via nuclear magnetic resonance and liquid chromatography-tandem mass spectrometry analyses for various compounds, including SCFAs, organic acids, and peptides. Several metabolites were found to be increased compared to the negative control. The resulting postbiotics were freeze-dried and stored at −20 °C until use.

### Mice

Eight-week-old male C57BL/6 mice were purchased from Orient Bio Inc. (Seongnam, Korea). The animals were maintained under specific-pathogen-free conditions at the Institute of Medical Science of the Catholic University of Korea under controlled temperature (21–22 °C) and light (12/12 h light/dark cycle) with standard mouse chow and water. All experimental procedures were approved by the Department of Laboratory Animals Institutional Animal Care and Use Committee of the School of Medicine, Catholic University of Korea, and conformed with all United States National Institutes of Health guidelines (Permit number: CUMC 2023-0232-02).

### Induction of colitis and drug administration

Acute colitis was induced by oral administration of 2% DSS (#02160110; Lot. YD05012; molecular weight 36,000-50,000; MP Biomedicals, Santa Ana, CA, USA) in distilled water offered *ad libitum* for 5 days, after which regular drinking water was provided. Mice were stratified based on their individual body weight to ensure comparable mean weights across all experimental groups, and randomly assigned to their respective treatment groups. The normal control group consisted of three animals (n = 3), while the DSS control, probiotic, and postbiotic groups were initially allocated five animals each (n = 5). Due to unexpected mortality during the experimental period, the final number of animals in some treatment groups was reduced to four (n = 4). The postbiotics were administered at a dose of 2 × 10^8^ colony-forming unit (CFU) as previously described (27, 28). *L. paracasei* DCF0420, *L. plantarum* DCF0514, or *L. curvatus* DCF0620 (each at 2 × 10^8^ CFU in phosphate-buffered saline (PBS, #21600010; Thermo Fisher Scientific) were orally administered to mice from 7 days before DSS injection until the end of the experiment. To evaluate the *in vivo* efficacy of postbiotics, acute colitis was induced in C57BL/6 mice using the same DSS protocol. A volume of 200 μL of *L. curvatus* DCF0620 postbiotics (100 mg/mL in PBS) was orally administered from 7 days before DSS injection until the end of the experiment. During the experimental period, weight changes and the disease activity index (DAI) were monitored to assess disease severity. DAI was calculated based on weight loss, stool consistency, and visible gross bleeding, following a previously reported method (29). To ensure objectivity and rigor, the individuals responsible for DAI assessment and subsequent histological scoring were blinded to the treatment groups.

### Microbiome analysis of mouse feces

Fecal samples from mice were suspended in PBS and centrifuged at 11,000 × g for 1 min. Bacterial genomic DNA was extracted using the genomic deoxyribonucleic acid (DNA) Prep Kit (SolGent, Korea) following the manufacturer’s protocol. Extracted genomic DNA was quantified and normalized to a uniform concentration across all samples prior to library preparation. Microbial composition was assessed via tag-encoded 16S rRNA gene sequencing on the Illumina MiSeq platform (Illumina, San Diego, CA, USA). The V3–V4 region of the 16S rRNA gene was amplified using primers 341F (5′-CCTACGGGNGGCWGCAG-3′) and 785R (5′-GACTACHVGGGTATCTAATCC-3′) (30). Raw sequence reads were trimmed with Cutadapt and processed using the Deblur pipeline in QIIME 2 (31, 32). Taxonomic classification was performed using a Naive Bayes classifier trained on the SILVA 132 database, with weighted classification to enhance accuracy. Alpha diversity metrics were calculated using QIIME 2.

### Histopathological analysis

Colon tissues were fixed in 10% (v/v) neutral-buffered formalin (#HT501320, Sigma-Aldrich) and sectioned at a thickness of 5 μm. The sections were stained with hematoxylin and eosin (#ab220365, Abcam) and examined via photomicroscopy (Olympus, Tokyo, Japan) at 40 × or 200 × magnification, using a scoring system based on four histological parameters (33). Epithelial loss was scored as follows: 0, no loss; 1, 0–5%; 2, 5–10%; 3, > 10%. Crypt damage was scored as: 0, none; 1, 0–10% loss; 2, 10–20% loss; 3, > 20% loss. Goblet cell depletion was scored as: 0, none; 1, mild; 2, moderate; 3, severe. Inflammatory cell infiltration was scored as: 0, none; 1, mild; 2, moderate; 3, severe. Scoring was performed independently by two blinded observers, and total scores were calculated by summing the individual parameter scores. Masson’s trichrome staining was conducted using a commercial kit (#25088–100, Polysciences, Warrington, PA, USA) to assess collagen deposition in colon tissues. Stained sections were examined via photomicroscopy (Olympus) at 200× magnification.

### Immunohistochemistry

Colon sections were analyzed immunohistochemically using the Envision Detection kit (#5007; DAKO, Glostrup, Denmark). The sections were incubated with primary antibodies (Abs) against TNF-α (#ab6671; Abcam, Cambridge, UK), IL-1β (#ab9722; Abcam), IL-6 (#ab7737; Abcam), IL-17 (#ab79056; Abcam), transforming growth factor (TGF)-β (#BS-0086R; Bioss, Woburn, MA, USA), type I collagen (Col1) (#ab6308; Abcam), and α-smooth muscle actin (α-SMA) (#ab7817; Abcam) for 2 h at room temperature. Then, they were incubated with a EnVision Detection SystemsPeroxidase/DAB (#K5007, Dako) for 30 min. The final products were developed using the DAB plus substrate system (#k346811-2, Dako). Immunostained sections were examined via photomicroscopy (Olympus). The numbers of positive cells were counted in high-power digital images (magnification: 400 ×) using Adobe Photoshop software (Adobe, San Jose, CA, USA). Positive cells were visually enumerated by three individuals, and the mean values were calculated.

### Isolation and stimulation of splenocytes

Murine splenocytes were prepared as previously described (34). Spleens were homogenized using sterilized glass slides with frosted ends, and red blood cells were lysed in hypotonic ammonium-chloride-potassium buffer (0.15 mM NH_4_Cl, 1 mM KCO_3_, and 0.1 mM EDTA, pH 7.4). The remaining splenocytes were filtered through a 40-µm cell strainer (#93040, SPL, Korea) and maintained in Roswell Park Memorial Institute (RPMI) 1640 medium (#31800089, Thermo Fisher Scientific) supplemented with 5% fetal bovine serum (#16000044, Thermo Fisher Scientific). The cells were pretreated with 0.1, 1, or 10 μg/mL *L. paracasei* DCF0420, *L. plantarum* DCF0514, or *L. curvatus* DCF0620 for 2 h. Following pretreatment, splenocytes were cultured for 72 h with either 0.5 μg/mL anti-CD3 Abs (#553057; BD Pharmingen, Franklin Lakes, NJ, USA) or 100 ng/mL lipopolysaccharide (LPS; #L4391; Sigma-Aldrich).

### Enzyme-linked immunosorbent assay

The levels of interferon (IFN)-γ, IL-17, and IL-10 in culture supernatants were determined using a sandwich enzyme-linked immunosorbent assay (ELISA) (#DY421; R&D Systems, Minneapolis, MN, USA). 96-well plates were coated with anti-mouse IFN-γ, IL-17, and IL-10 capture Ab and incubated overnight at 4 ˚C temperature. After the overnight incubation, the plates were blocked with 200 μL PBS containing 1% bovine serum albumin (#A7030, Merck) and 0.05% Tween 20 (#P1379, Sigma-Aldrich) for 2 h at room temperature. Mouse IFN-γ, IL-17, and IL-10 standards were diluted twofold from 1000 to 15.6 pg/mL. Culture supernatant or standards in reagent diluent were added to the plates and incubated at room temperature for 2 h. The cytokine concentrations were reported as the absolute amount in the supernatant (pg/mL) based on the standard curve, and were not normalized by cell number. Subsequently, the plates were washed, and 50 μL of detection Ab, diluted in reagent diluent, was added to each well, and incubated for 2 h at room temperature. The plates were washed and then 50 μL of working dilution of Streptavidin-HRP B was added to each well, and incubated for 20 min at room temperature. The plates were washed and then 50 μL of substrate solution was added to each well, and incubated for 20 min at room temperature. 50 μL of stop solution was added to each well and absorbance at 450 nm was measured on an ELISA microplate reader (Molecular Devices, San Jose, CA, USA).

### CCD-18Co culture

CCD-18Co cells (Korean Cell Line Bank), a non-malignant fibroblast cell line isolated from normal human colon tissue, were pretreated with 10 μg/mL *L. paracasei* DCF0420, *L. plantarum* DCF0514, or *L. curvatus* DCF0620 for 2 h. Then, the cells were stimulated with 10 ng/mL TGF-β (#100-21C-10; Peprotech, Cranbury, NJ, USA) for 24 h to obtain protein from the cells.

### Immunoblot analysis

Cells were lysed in Halt protein lysis buffer containing Halt phosphatase inhibitor (#78440; Thermo Fisher). Lysates were centrifuged at 14,000 × *g* for 15 min at 4 °C. Protein concentrations were determined using the Bradford protein assay (#23225, Thermo Fisher Scientific). Proteins were electrophoretically separated via sodium dodecyl sulfate–polyacrylamide gel electrophoresis and transferred to Hybond enhanced chemiluminescence membranes (#10600001; Cytiva, Marlborough, MA, USA). Membranes were incubated with Abs against fibronectin (#ab2413; Abcam) and β-actin (#SC-47778; Santa Cruz Biotechnology, Santa Cruz, CA, USA). Hybridized bands were detected using an ECL detection kit (#RPN2106, Merck) and X-film (#EA8EC, Agfa, Mortsel, Belgium). Western blotting analysis was performed using the SNAP i.d. Protein Detection System (Millipore, Burlington, MA, USA).

### Statistical analysis

All statistical analyses were performed using GraphPad Prism (v4 for Windows; GraphPad Software, Inc., La Jolla, CA, USA). Data are presented as means ± standard deviations (SDs). Statistical comparisons between two groups were performed using the parametric Student’s *t*-test. Statistical analysis among three or more groups was performed using one-way ANOVA followed by Tukey’s honestly significant difference test for *post-hoc* multiple comparisons. *P*-values < 0.05 (two-tailed) were considered statistically significant.

## Results

### *L. paracasei* DCF0420, *L. plantarum* DCF0514, and *L. curvatus* DCF0620 improve inflammation and fibrosis *in vitro*

**Figure 1:**
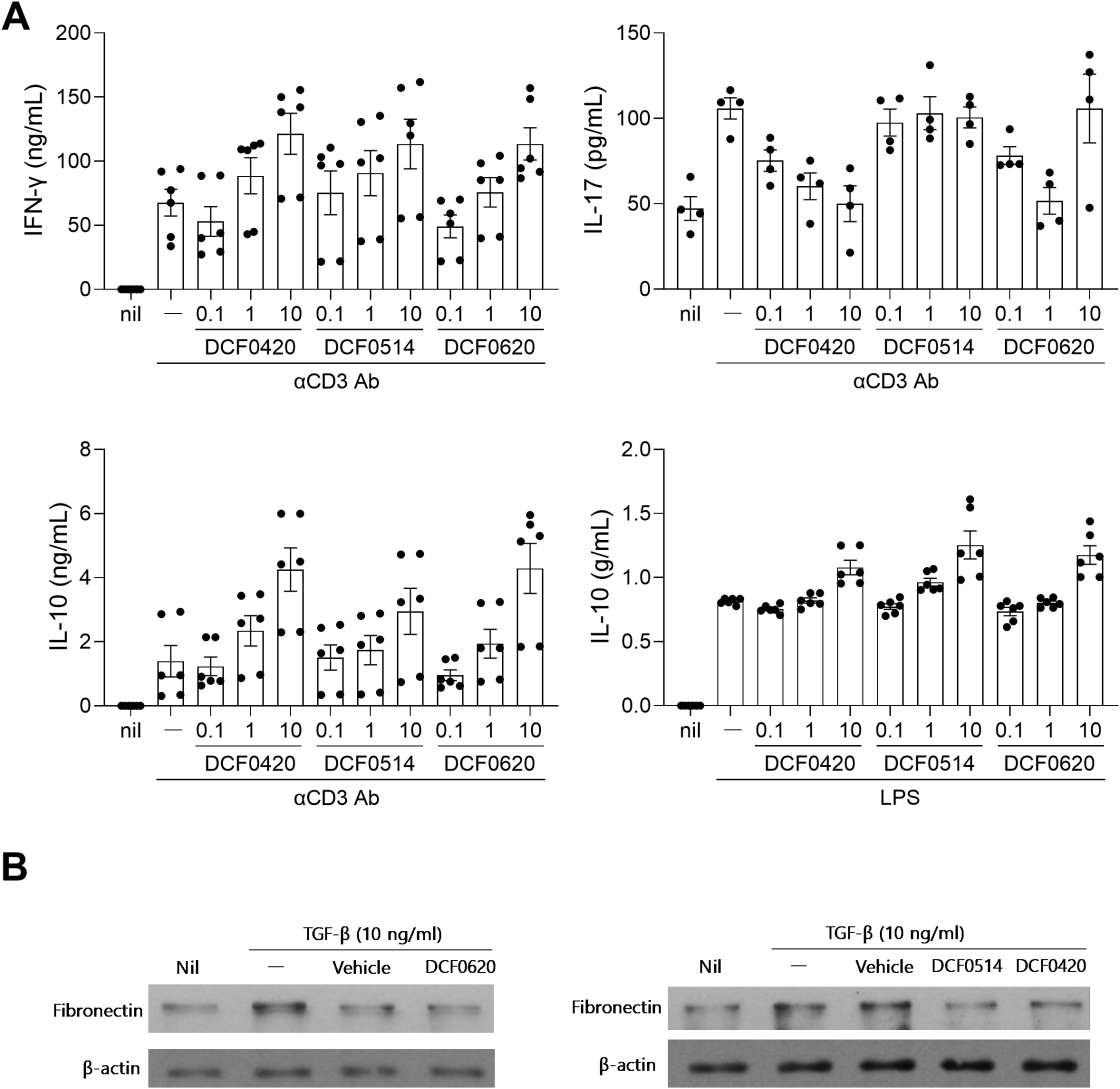
*L. paracasei* DCF0420, *L. plantarum* DCF0514, and *L. curvatus* DCF0620 improve inflammation and fibrosis *in vitro*. **(A)** Splenocytes from C57BL/6 mice (n = 4-6) were stimulated with each strain separately (at 0.1, 1, or 10 μg/mL) or vehicle (saline) for 2 h and then cultured with anti-CD3 Ab (2 μg/mL) or LPS (500 ng/mL) for 3 days. The levels of IFN-γ, IL-17 and IL-10 in the culture supernatant were determined via ELISA. **(B)** CCD-18Co cells were treated with each strain separately (at 10 μg/mL) or vehicle (saline) in the presence of TGF-β (10 ng/mL) for 24 h. The levels of fibronectin were assessed via immunoblotting. Values are presented as means ± SDs. Data are representative of two independent experiments.

To determine the effect of each probiotic on the regulation of immune cells *in vitro*, murine splenocytes were cultured with *L. paracasei* DCF0420, *L. plantarum* DCF0514, or *L. curvatus* DCF0620 in the presence of anti-CD3 Ab or LPS for 3 days. These three probiotics enhanced the production of IFN-γ in a dose-dependent manner under anti-CD3 Ab stimulation. Additionally, they enhanced the production of the anti-inflammatory cytokine IL-10 in a dose-dependent manner under anti-CD3 Ab or LPS stimulation (**Figure 1A**). To investigate their effects on fibrosis, CCD-18Co cells, human fibroblast cell line isolated from normal colon tissue, were cultured in the presence of TGF-β and treated with each probiotic. The level of fibronectin, an ECM protein synthesized in activated myofibroblasts, was reduced by treatment with each probiotic; DCF0620 had the greatest effect (**Figure 1B**). These results indicate that *L. paracasei* DCF0420, *L. plantarum* DCF0514, and *L. curvatus* DCF0620 have the potential to alleviate inflammation and fibrosis *in vitro*.

### *L. curvatus* DCF0620 reduces the severity of DSS-induced colitis

**Figure 2:**
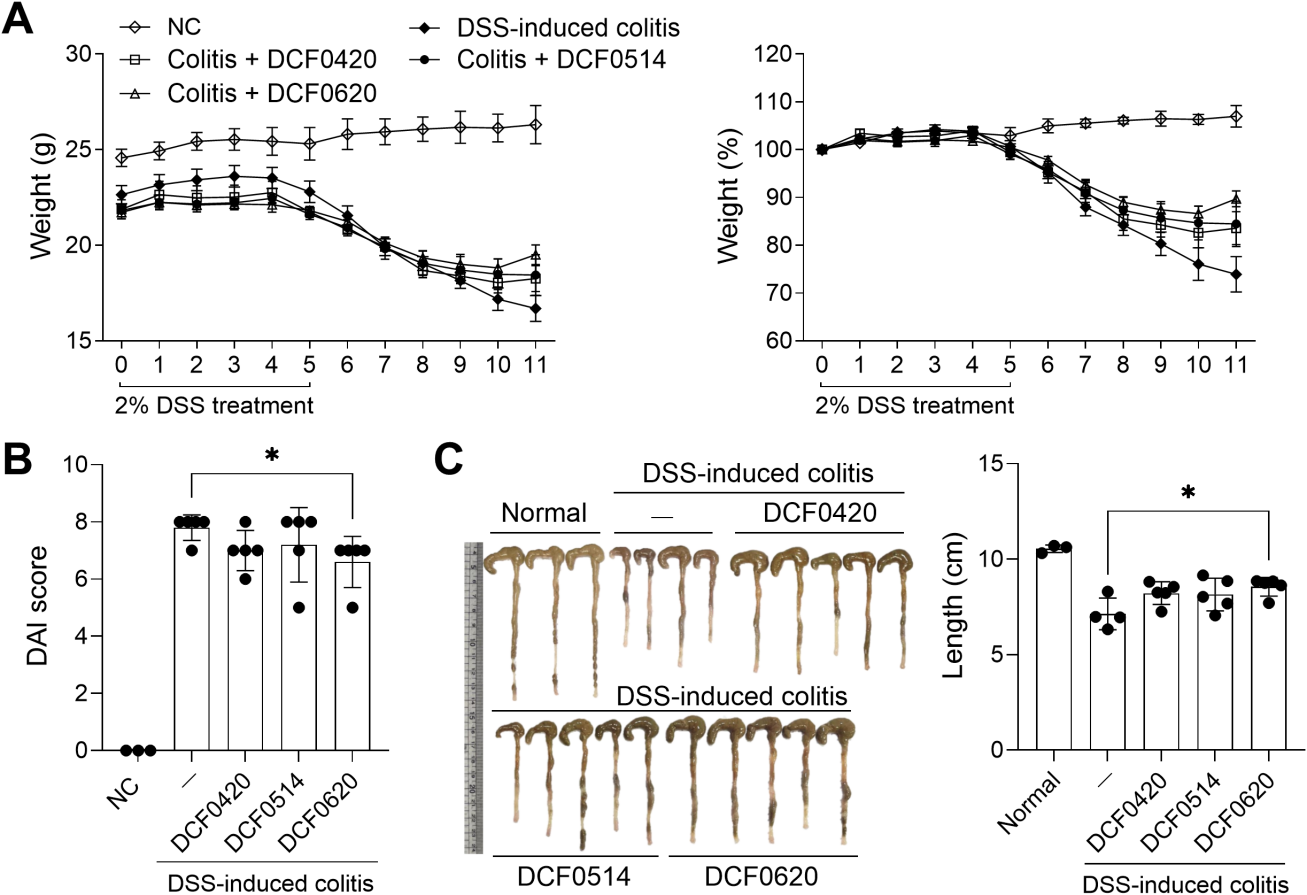
*L. curvatus* DCF0620 effectively reduces the clinical symptoms of DSS-induced colitis. Acute colitis was induced in C57BL/6 mice by oral administration of 2% DSS. Then *L. paracasei* DCF0420, *L. plantarum* DCF0514, or *L. curvatus* DCF0620 (2 × 10^8^ CFU in PBS) was orally administered from 7 days before DSS injection until the end of the experiment (n = 4-5/group, normal control n = 3). **(A)** Changes in body weight were measured, and the percentage change was calculated based on the initial weight (at day 0). **(B)** DAI score on day 11 after DSS administration. **(C)** Colon lengths were assessed on day 11 after DSS administration. Changes in colon length were measured. Data are expressed as mean ± SDs. Statistical analysis was performed using One-way ANOVA followed by Tukey’s test. *P < 0.05 vs. colitis control group.

To investigate the efficacy of each probiotic in improving the development of IBD, *L. paracasei* DCF0420, *L. plantarum* DCF0514, or *L. curvatus* DCF0620 was administered daily orally for 7 days prior to 2% DSS administration, and body weight changes were assessed. Compared with the control group, the DCF0620 group showed attenuated weight loss, decreased DAI (P = 0.0278), and improved colon shortening (P = 0.0147) (**Figure 2**).

### Each probiotic effectively suppresses intestinal inflammation in mice with colitis

**Figure 3:**
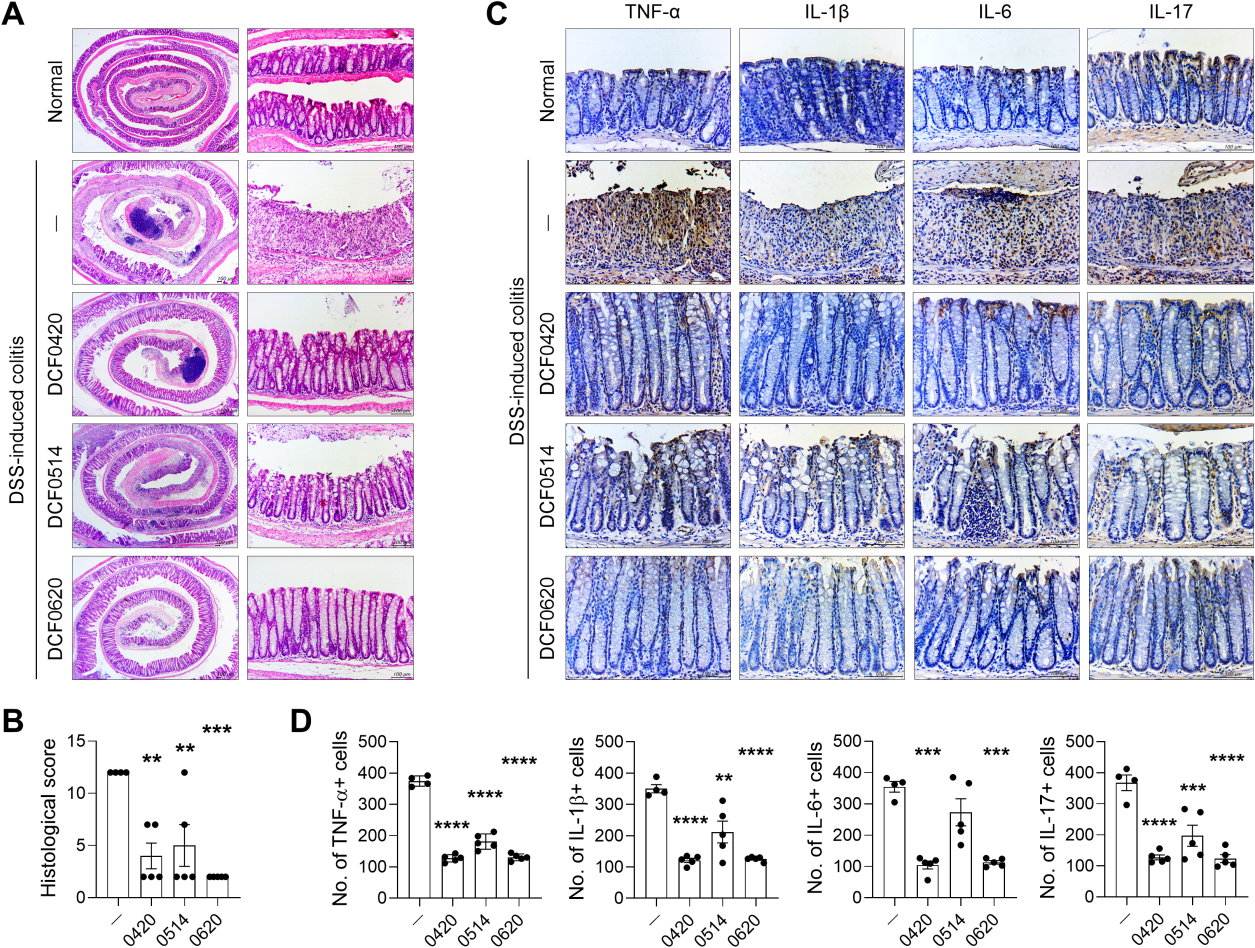
*L. paracasei* DCF0420, *L. plantarum* DCF0514, and *L. curvatus* DCF0620 reduce colonic inflammation in mice with colitis. Acute colitis was induced in C57BL/6 mice by oral administration of 2% DSS. Then, each strain was orally administered to the mice (2 × 10^8^ CFU in PBS) from 7 days before DSS injection until the end of the experiment (n = 4-5/group, normal control n = 3). **(A, B)** On day 11, colon tissue sections were stained with hematoxylin and eosin. Representative images are shown (original magnification: 200×, scale bar: 100 µm). The graph displays the histological scores **(B)**. **(C, D)** On day 11, colon tissue sections were stained with Abs against TNF-α, IL-1β, IL-6, and IL-17. Representative images are shown (original magnification: 400×, Scale bar: 100 µm). Graphs display the numbers of Ab-positive cells per field **(D)**. Data are expressed as mean ± SDs. Statistical analysis was performed using One-way ANOVA followed by Tukey’s test. **P < 0.01, ***P < 0.001, ****P < 0.0001.

To evaluate the therapeutic effects of each probiotic, we assessed inflammatory cell infiltration and intestinal tissue damage across the experimental groups. All probiotics effectively mitigated colon damage, including inflammatory cell infiltration, crypt loss, and ulceration, compared to the control group (DCF0420 P = 0.0017; DCF0514 P = 0.0057; DCF0620 P = 0.0002) (**Figure 3**). To further investigate their anti-inflammatory potential, we performed immunohistochemical analysis to detect cells expressing inflammatory cytokines within intestinal tissue sections. Mice with colitis exhibited a marked increase in the infiltration of cytokine-expressing cells relative to normal mice. In contrast, mice treated with each probiotic showed a significant reduction in the infiltration of cells expressing inflammatory cytokines compared to untreated colitis controls (**Figure 3**). Specifically, the infiltration of TNF-α-expressing cells was significantly reduced across all probiotic-treated groups (P < 0.0001). Similarly, IL-1β expression was significantly decreased following treatment (DCF0420 P < 0.0001; DCF0514 P = 0.0026; DCF0620 P < 0.0001). For IL-6 significant reductions were observed in DCF0420 (P = 0.0001) and DCF0620 (P = 0.0002) treated mice. Furthermore, a highly significant reduction was noted in IL-17-expressing cells across all three probiotic groups (DCF0420 P < 0.0001; DCF0514 P = 0.0001; DCF0620 P < 0.0001) compared to untreated colitis controls. These findings indicate that each probiotic strain can attenuate intestinal inflammation by suppressing the infiltration of inflammatory cells.

### Each probiotic effectively suppresses intestinal fibrosis in mice with colitis

**Figure 4:**
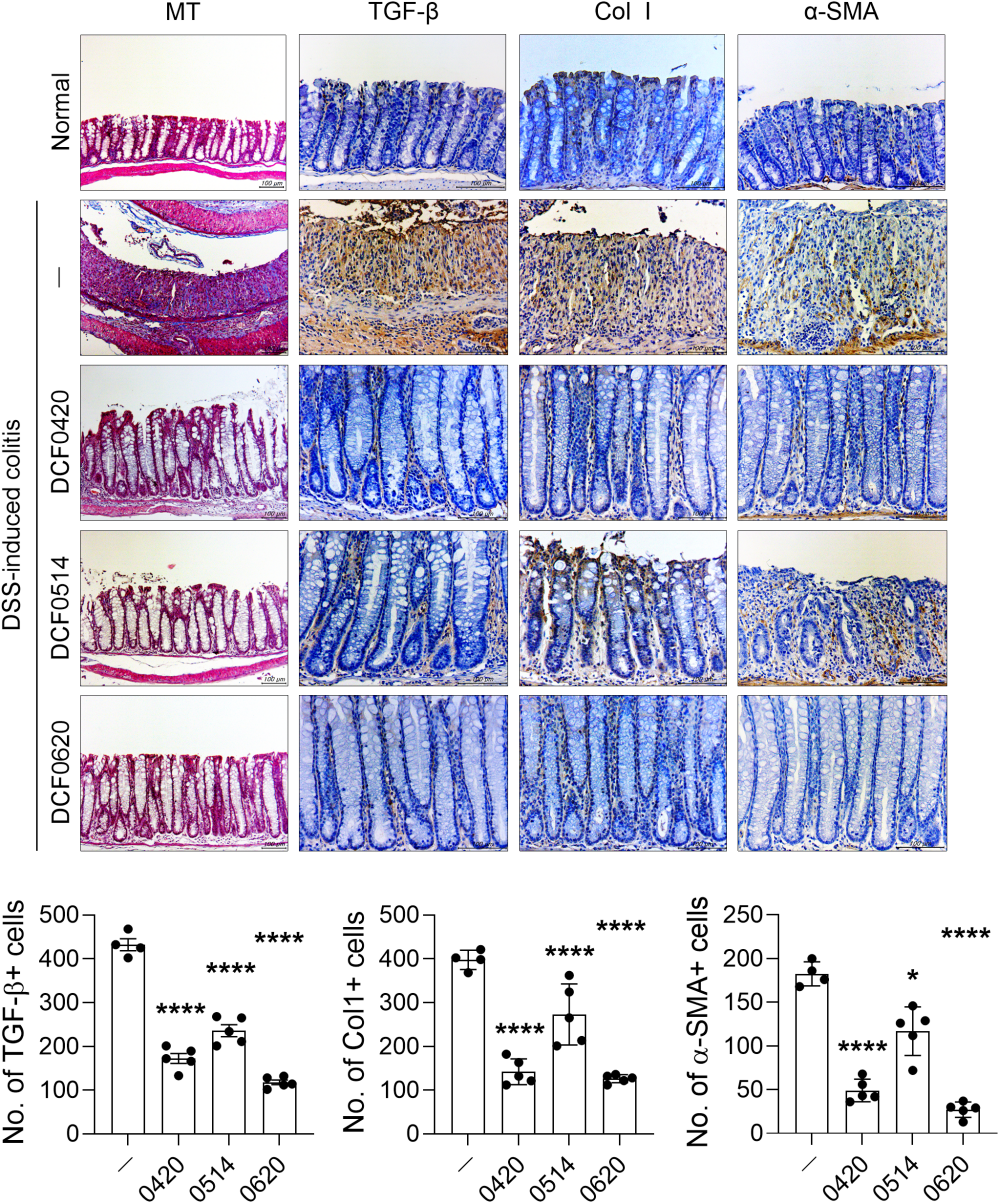
*L. paracasei* DCF0420, *L. plantarum* DCF0514, and *L. curvatus* DCF0620 reduce colonic fibrosis in mice with colitis. Acute colitis was induced in C57BL/6 mice by oral administration of 2% DSS. Each strain was orally administered to the mice from 7 days before DSS injection until the end of the experiment (n = 4-5/group, normal control n = 3). On day 11, colon tissue sections were stained with Masson’s trichrome and Abs against TGF-β, Col1, and α-SMA. Representative images are shown (original magnification: 400×, Scale bar: 100 µm). Graphs display the numbers of Ab-positive cells per field. Data are expressed as mean ± SDs. Statistical analysis was performed using One-way ANOVA followed by Tukey’s test. *P < 0.05, ****P < 0.0001.

In addition, each probiotic exhibited antifibrotic effects, as demonstrated by Masson’s trichrome staining. To assess fibroblast differentiation, immunohistochemistry was performed on intestinal tissue sections from mice treated with each probiotic. The infiltration of cells expressing TGF-β, a key regulator of fibrosis (35), was notably reduced in the probiotic-treated groups compared to controls (P < 0.0001 for all strains). Similarly, the infiltration of cells expressing Col1 (P < 0.0001 for all strains) and α-SMA treatment (DCF0420 P < 0.0001; DCF0514 P = 0.0455; DCF0620 P < 0.0001), markers of activated myofibroblasts (36), was diminished in the intestinal tissues of probiotic-treated mice (**Figure 4**). These findings suggest that each probiotic may modulate the differentiation of fibroblasts into myofibroblasts, thereby contributing to the mitigation of fibrotic progression in chronic colitis.

### Postbiotics from *L. curvatus* DCF0620 ameliorate disease development

**Figure 5:**
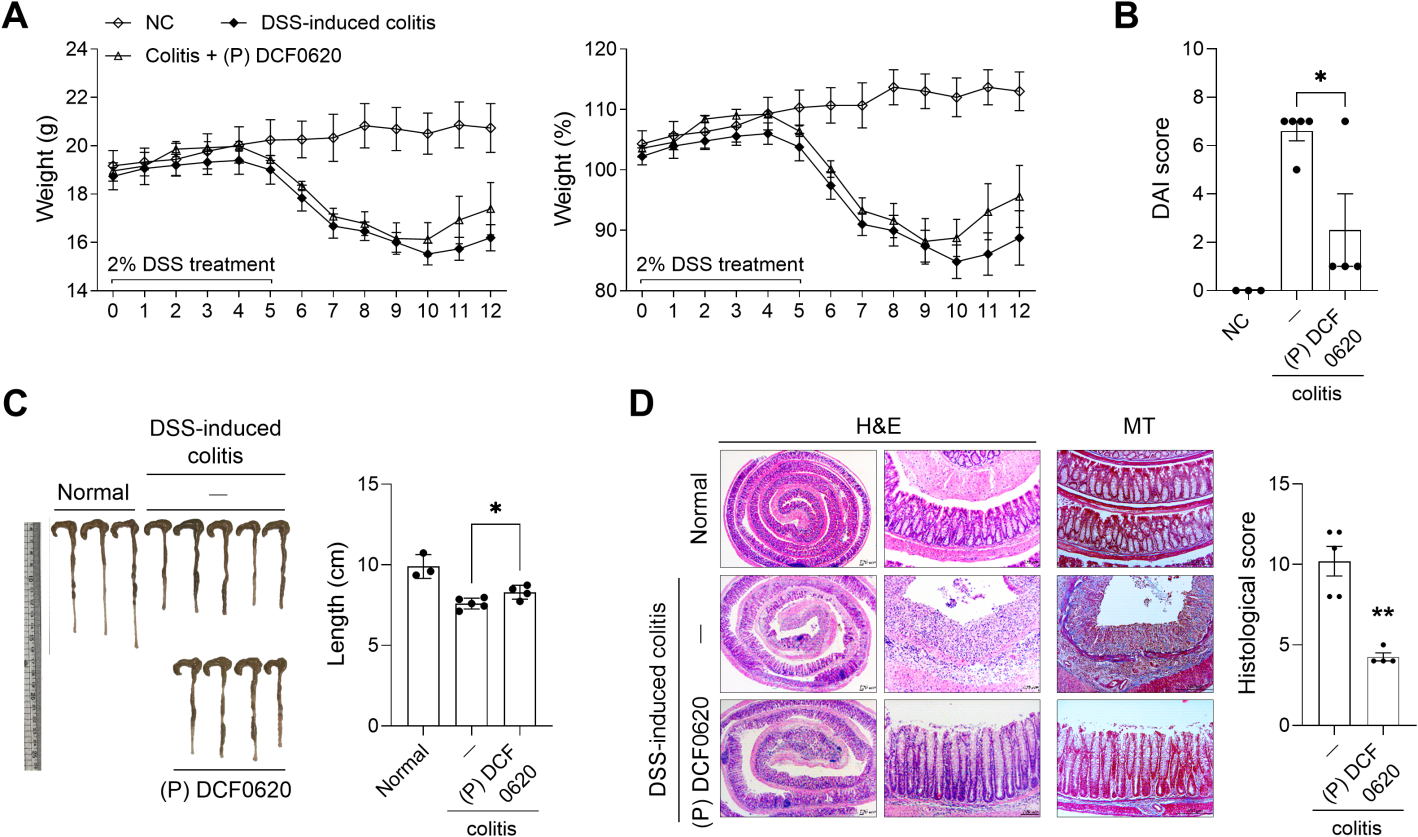
Postbiotics from *L. curvatus* DCF0620 effectively reduces the clinical symptoms of colitis. Acute colitis was induced in C57BL/6 mice by oral administration of 2% DSS. Then, 200 μL DCF0620 postbiotics (100 mg/mL in PBS) was orally administered to the mice from 7 days before DSS injection until the end of the experiment (n = 4-5/group, normal control n = 3). **(A)** Changes in body weight were measured, and the percentage change was calculated based on the initial weight (at day 0). **(B)** DAI score on day 12 after DSS administration. **(C)** Colon lengths assessed on day 12 after DSS administration. **(D)** On day 12, colon tissue sections were stained with hematoxylin and eosin and Masson’s trichrome. Representative images are shown (original magnification: 200×, scale bar: 100 µm). The graph displays the mean histological scores. Data are expressed as mean ± SDs. Statistical analysis was performed using One-way ANOVA followed by Tukey’s test. *P < 0.05, **P < 0.01.

Next, to investigate the therapeutic efficacy of *L. curvatus* DCF0620 postbiotics in mice with colitis, we isolated the postbiotics after culturing *L. curvatus* DCF0620 in soy germ milk. We administered them orally daily for 7 days prior to 2% DSS injection. Compared to controls, weight loss was alleviated in mice given postbiotics; in addition, their DAI was significantly lower (P = 0.0216) and colon shortening (P = 0.0282) was reduced (**Figures 5A-C**). Compared to the control group, the group administered postbiotics exhibited alleviation of intestinal structural damage, reduced inflammatory cell infiltration, and, notably, decreased collagen deposition within the colonic tissue (P = 0.0015) (**Figure 5D**). These results demonstrate that *L. curvatus* DCF0620 postbiotics also possess therapeutic efficacy against colitis.

### Postbiotics from *L. curvatus* DCF0620 reduce intestinal fibrosis and inflammation

**Figure 6:**
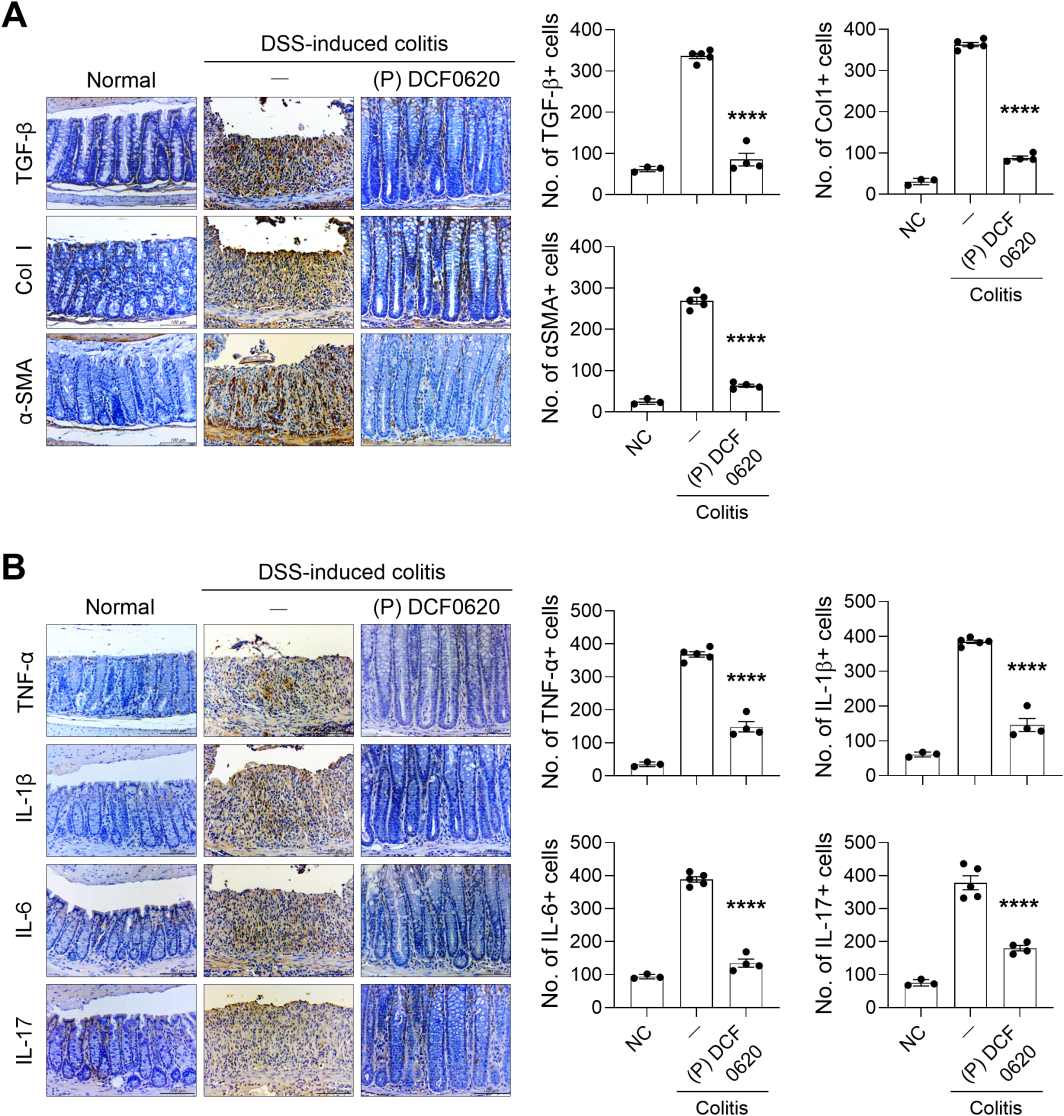
Postbiotics from *L. curvatus* DCF0620 reduce colonic fibrosis and inflammation in mice with colitis. Acute colitis was induced in C57BL/6 mice by oral administration of 2% DSS. Then, 200 μL of postbiotics (100 mg/mL in PBS) was orally administered to the mice from 7 days before DSS injection until the end of the experiment (n = 4-5/group, normal control n = 3). **(A)** On day 12, colon tissue sections were stained with Abs against TGF-β, Col1, and α-SMA. **(B)** On day 12, the sections were stained with Abs against TNF-α, IL-1β, IL-6, and IL-17. Representative images are shown (original magnification: 400×, Scale bar: 100 µm). Graphs display the numbers of Ab-positive cells per field. Data are expressed as mean ± SDs. Statistical analysis was performed using One-way ANOVA followed by Tukey’s test. ****P < 0.0001.

To determine whether postbiotics from *L. curvatus* DCF0620 also have antifibrotic effects, we examined the number of cells expressing fibrosis-related factors in intestinal tissue. Compared to controls, the infiltration of cells expressing TGF-β (P < 0.0001), Col1 (P < 0.0001), α-SMA (P < 0.0001), TNF-α (P < 0.0001), IL-1β (P < 0.0001), IL-6 (P < 0.0001), or IL-17 (P < 0.0001) was reduced in the intestinal tissues of mice given the postbiotics (**Figure 6**). These results demonstrate that the postbiotics can effectively control intestinal fibrosis and inflammatory responses in mice with colitis.

### Postbiotics from *L. curvatus* DCF0620 show protective effects against dysbiosis

**Figure 7:**
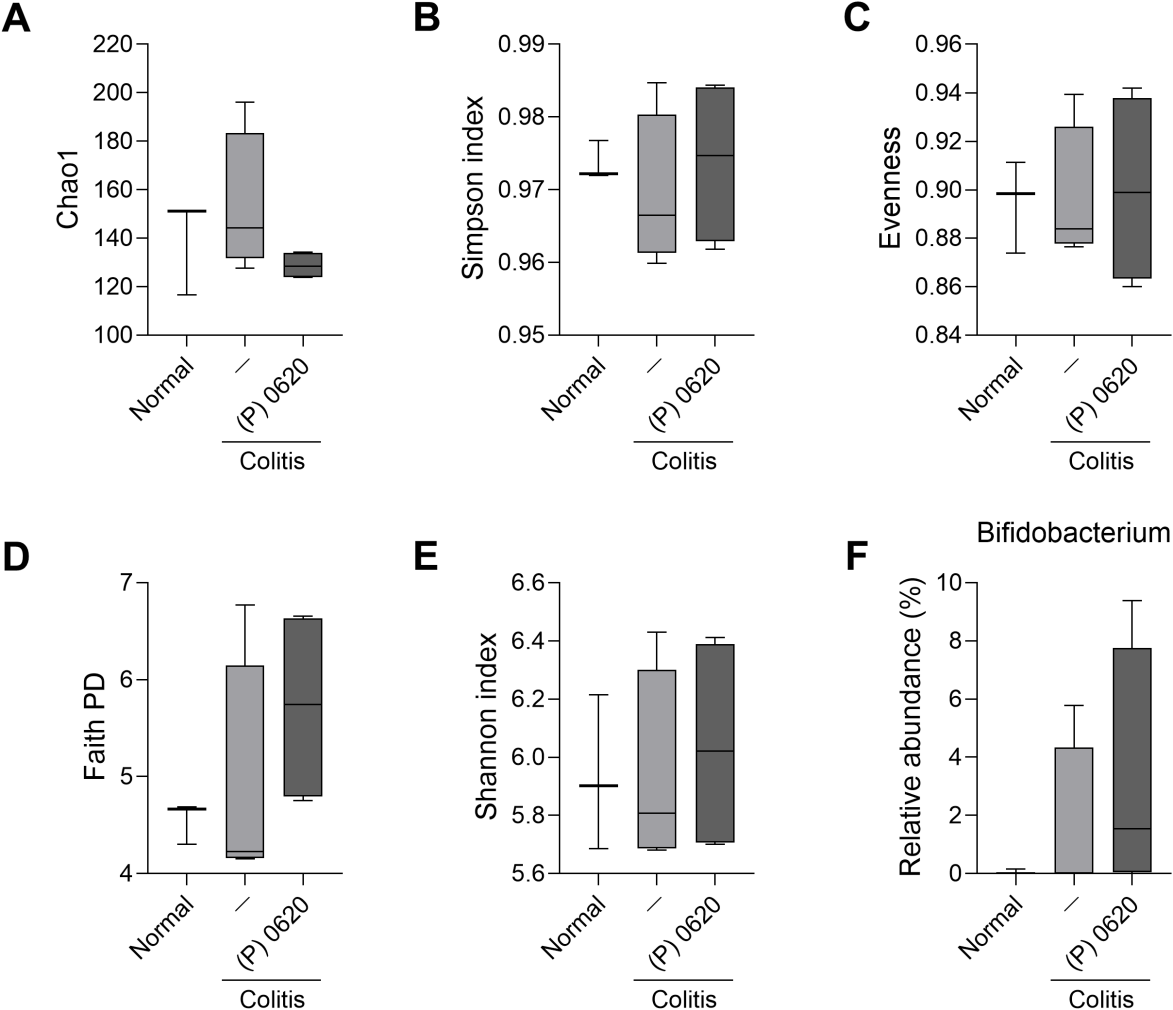
Effects of postbiotics from *L. curvatus* DCF0620 on the diversity and composition of the gut microbiota. Acute colitis was induced in C57BL/6 mice by oral administration of 2% DSS. Then, 200 μL of postbiotics (100 mg/mL in PBS) was orally administered to the mice from 7 days before DSS injection until the end of the experiment (n = 4-5/group, normal control n = 3). On day 12, fecal samples were collected from the mice in each group and stored at –80°C prior to 16S rRNA gene sequencing. **(A–E)** Alpha diversity indices. **(A)** Chao1 index, **(B)** Simpson index, **(C)** Evenness index, **(D)** Faith’s phylogenetic diversity, and **(E)** Shannon’s index. **(F)** Relative abundance of *Bifidobacterium*. Data are expressed as mean ± SDs.

Next, we analyzed the effects of these postbiotics on the gut microbiota. All samples had >20,000 high-quality reads after filtering, and the number of observed ASVs reached saturation at this depth (**Supplementary Figure S1** in the Supplementary Material). The Chao1 index, (**Figure 7A**), an indicator of species richness within a community, showed no significant differences between the postbiotic groups and normal mice. The Simpson (**Figure 7B**) and Shannon (**Figure 7E**) indices, indicators of species diversity within a community, showed that microbiota diversity was lower in mice with colitis versus controls. However, those given the postbiotics had higher indices. The colitis group also had lower Faith’s PD values (**Figure 7D**), an indicator of phylogenetic diversity, but mice given the postbiotics had higher values. Evenness values (**Figure 7C**), which indicate the degree of uniformity in the relative abundance of each species within a community, tended to be lower in mice with colitis, while those given the postbiotics had higher values. The relative abundance of *Bifidobacterium* (**Figure 7F**), a representative probiotic strain with known benefits for gut health (37), was higher in mice administered the postbiotics compared to those with colitis who were left untreated (**Figure 7**). These results demonstrate that the postbiotics from *L. curvatus* DCF0620 significantly attenuates the decrease in microbial diversity and contributes to the improvement of the impaired microbiota balance of mice with colitis, while increasing the relative abundance of *Bifidobacterium*, a beneficial bacterium.

## Discussion

We compared the anti-IBD modulating effects of *L. paracasei* DCF0420, *L. plantarum* DCF0514, and *L. curvatus* DCF0620, isolated from traditional Korean fermented foods. *In vitro*, all three strains dose-dependently increased the levels of the anti-inflammatory cytokine IL-10 in murine splenocytes and reduced the levels of fibronectin, a pro-fibrotic factor, in fibroblasts. Consistent with previous reports, these strains all exhibited therapeutic effects in mice with colitis (22, 38, 39). Among these, *L. curvatus* DCF0620 demonstrated the strongest anti-inflammatory and anti-fibrotic effects. To investigate the colitis-modulating efficacy of postbiotics derived from this strain, we cultured it with soy germ milk and administered the postbiotics into mice with colitis. This treatment effectively reduced disease severity and improved gut microbiota imbalance.

Probiotics play a beneficial role in maintaining immune defense, suppressing intestinal pathogens, and improving epithelial cell barrier function by regulating the balance of the intestinal microflora (40, 41). LAB, in particular, are generally recognized as safe food-grade microorganisms (41). LAB produce primary SCFAs (42), including acetate, butyrate, and propionate, through the fermentation of dietary fiber (43). These metabolites regulate intestinal immunoglobulin A levels, modulate mucosal and systemic immune responses, and support epithelial barrier function, contributing to the alleviation of various inflammatory diseases, including IBD (44–46).

Previous studies have reported the disease-modifying effects of *L. paracasei* and *L. plantarum* in mouse models of colitis (38, 47, 48) but few studies have examined the effects of *L. curvatus* on this disease. Wang et al. reported that *L. curvatus* restores intestinal length and improves the DAI in mice with colitis (22). We found that *L. paracasei* DCF0420, *L. plantarum* DCF0514, and *L. curvatus* DCF0620 all reduced colitis severity, with DCF0620 exhibiting the most effective anti-inflammatory, anti-fibrotic, and disease-modifying effects. Previous research demonstrated that *L. curvatus* isolated from kimchi increases IL-10 production in mouse bone marrow-derived dendritic cells through the activation of the nuclear factor kappa-light-chain-enhancer of activated B cells (NFκB) and extracellular signal-regulated kinase (ERK) signaling pathways (21). In our study, we confirmed that *L. curvatus* DCF0620 significantly increased IL-10 production in splenocytes under both T cell activation (anti-CD3 Ab) and innate cell/B cell activation (LPS) conditions. This suggests that DCF0620 promotes IL-10 production potentially involving various immune cell types. We observed increased *IL-10* mRNA levels, concurrent with increased expression of forkhead box P3 (*Foxp3*) mRNA, the master regulatory gene for regulatory T cells (49), in splenic tissues from DSS-induced colitis mice administered with DCF0620 (data not shown). While the precise source of IL-10 production requires further study using isolated cells, the *Foxp3* mRNA data suggest a strong possibility of the involvement of the regulatory T cells pathway.

Most probiotics are administered orally; thus, they pass through the stomach, limiting their ability to successfully reach the intestine (50, 51). Postbiotics, on the other hand, are more stable and safer because they can resist gastrointestinal stress and can be administered in precise doses (23, 52); they constitute a complex mixture of inactivated microorganisms, bacterial components, and metabolites secreted by probiotics (52, 53). Furthermore, unlike live probiotics, postbiotics are widely recognized to offer significant regulatory and commercial advantages. Because they are non-viable microbial components, postbiotics are reported to reduce the risks associated with infection and antibiotic resistance gene transfer. Their high stability facilitates standardization and long-term storage without refrigeration, making them essential for large-scale industrial and clinical applications (23). Therefore, we also investigated the therapeutic efficacy of postbiotics derived from *L. curvatus* DCF0620, the most effective of the three probiotics, in mice with colitis. They reduced disease severity, exhibiting anti-inflammatory and anti-fibrotic effects and improving intestinal dysbiosis. Since soy germ contains various bioactive compounds, we used a non-fermented soy germ extract as a vehicle control. The observed beneficial effects are therefore attributable to the metabolic conversion and postbiotic components produced by *L. curvatus* DCF0620 fermentation, rather than the intrinsic compounds of the soy germ. Comparative analysis of the α-diversity indices suggests that the observed recovery in α-diversity in the postbiotic-treated group is predominantly driven by the restoration of species richness, rather than evenness. While the Simpson and Shannon indices (**Figures 7B, E**) showed an increasing trend in the postbiotic-treated group compared to the colitis control group, the Evenness index (**Figure 7C**) showed no substantial difference. This suggests that the postbiotic treatment helped restore the diversity of bacterial species (richness) that were lost due to colitis, but the relative abundance equilibrium (evenness) among those species did not change substantially. This recovery in richness may reflect the potential restoration of various commensal bacteria lost due to colitis, which could contribute to enhanced resilience of the gut microbial ecosystem. Detailed analyses at the genus and family levels revealed that several beneficial gut taxa beyond *Bifidobacterium* were restored in the postbiotic-treated group. At the family level, the relative abundance of Lachnospiraceae and Bifidobacteriaceae, which are associated with SCFA production and intestinal mucosal homeostasis, increased compared to vehicle-injected colitis mice. Conversely, the abundance of Enterobacteriaceae, a bacterial family associated with inflammation, decreased. At the genus level, key beneficial genera (Lachnospiraceae NK4A136 group, *Roseburia*, *Ruminococcus*, *Faecalibacterium*, etc.), which were significantly reduced in the colitis group, increased after postbiotic treatment. Conversely, genera associated with inflammation, such as *Escherichia-Shigella* and *Clostridia* UCG-014, were reduced. These changes suggest that postbiotics contributed to the restoration of intestinal balance by promoting the recovery of SCFA-producing commensal bacteria and suppressing the proliferation of inflammation-associated bacteria.

Postbiotics are reported to have disease-improving effects similar to those of probiotics. Live *L. paracasei* and its postbiotics increase the relative abundance of beneficial bacteria and elevate SCFA levels in mice with colitis (54). Postbiotics from *Lacticaseibacillus rhamnosus* 1.0320 reduce colonic tissue damage and inflammatory cytokine secretion in such mice, attenuating disease progression (55). Similar findings have been reported for BLEPS-1, an exopolysaccharide derived from *Bifidobacterium longum* subsp. *longum* XZ01, together with *Lactobacillus acidophilus* (56). These results suggest that postbiotics exhibit comparable efficacy to probiotics, with diverse biological functions.

We compared the disease-improving effects of *L. paracasei* DCF0420, *L. plantarum* DCF0514, and *L. curvatus* DCF0620 in mice with DSS-induced colitis and found that *L. curvatus* DCF0620 showed superior efficacy. Postbiotics from this strain also exhibited disease-improving, anti-inflammatory, anti-fibrotic, and intestinal dysbiosis-modifying effects. These findings strongly support the potential of *L. curvatus* DCF0620 and its postbiotics as novel therapeutic candidates for IBD. While this study provides compelling evidence for the therapeutic efficacy of *L. curvatus* DCF0620 and its postbiotics in a mouse model, we acknowledge its limitations for potential human clinical applications. The results of this study are based on a chemically induced acute mouse colitis model, and thus the effective doses used may not be directly applicable to human clinical settings. While *L. curvatus* is generally recognized as safe, and postbiotics are inherently safer than live bacteria, this study did not include a systematic analysis of the host toxicity, long-term safety, or dose-response profile of the postbiotic formulation. Therefore, to determine whether *L. curvatus* DCF0620 or soybean germ-based postbiotics can be used as viable IBD treatments, further studies are needed to conduct toxicological evaluations and pharmacokinetic/pharmacodynamic (PK/PD) analyses.

## Data Availability

The original contributions presented in the study are included in the article/supplementary material. Further inquiries can be directed to the corresponding authors.
