# Supplementary material for: Latilactobacillus curvatus DCF0620 and postbiotics derived from soybean germ reduce colitis severity by modulating fibrosis and gut dysbiosis
Source: Front Immunol. 2026 Jan 6;16:1726298. doi: 10.3389/fimmu.2025.1726298 (PMC12815751; doi:10.3389/fimmu.2025.1726298)
Supplement: Supplementary file 1 [file Table1.docx]

Supplementary Figure 1

Rarefaction curves of 16S rRNA gene sequences showing the relationship between sequencing depth and observed OTUs for all samples.
